# Shared book reading and promoting social and emotional competences in educational settings: a narrative review

**DOI:** 10.3389/fpsyg.2025.1622536

**Published:** 2025-08-15

**Authors:** Rotem Schapira, Ilaria Grazzani

**Affiliations:** ^1^The Academic College Levinsky-Wingate, Tel Aviv University, Tel-Aviv, Israel; ^2^Lab-PSE, Department of Human Sciences for Education “R. Massa”, University of Milano-Bicocca, Milan, Italy

**Keywords:** shared book reading, social and emotional competence, interventions, toddlerhood, preschoolers

## Abstract

Over the last 20 years plenty of studies have shown the association between the development of social and emotional skills on the one hand, and wellbeing, peer popularity and school achievement on the other hand. Against this background, numerous intervention studies have been implemented especially in school contexts. Given the importance of an early enhancement of such skills, following a narrative review method we set out to examine the impact of shared book reading interventions on the social and emotional competence of children attending nursery and preschool settings. We conducted a comprehensive data search based on PubMed, Google Scholar, Scopus, and Web of Science. We identified 15 studies, five focusing on the nursery setting and ten to the preschool environment. We illustrated the main characteristics of the studies, highlighting the most significant results. In conclusion the paper both provides some considerations on the reviewed studies and suggests future lines for research.

## 1 Introduction

Over the last decades numerous studies have shown the association between the development of social and emotional abilities on the one hand, and wellbeing, peer popularity and school achievement on the other hand (Denham, 2023). Against this background, plenty of intervention studies have been implemented especially in school contexts. Given the importance of an early enhancement of such skills, some researchers have turned their attention to the implementation of early interventions with toddlers and preschoolers. On this backdrop, in this article we review intervention studies conducted in nursery and preschool contexts that utilized shared book reading (SBR) to promote children’s social and emotional competences (SEC). To this end, we adopted the narrative review method, valued for its flexibility and practicality in deriving a comprehensive summary and critical appraisal of the scientific literature on a given topic (Sukhera, 2022), especially when the research field is not widespread and the study is exploratory. Research on SBR interventions in nursery and preschool settings, particularly those focusing on emotions, constitutes a recently emerging line of inquiry. Indeed, most studies on shared book reading have been implemented in home environments, targeting parents and primarily focusing on the impact of SBR on children’s language and literacy abilities (Mol et al., 2008).

We chose to focus exclusively on SBR interventions because SBR is a naturally embedded, developmentally appropriate practice that occurs daily in early childhood settings. Unlike other socio-emotional interventions that often require separate time allocations, special curricula, or external facilitators, SBR offers a readily accessible platform that can be seamlessly integrated into educational routines. In addition to its well-documented contributions to language and literacy development, SBR provides a rich context for emotional learning, offering children opportunities to explore characters’ feelings, engage in perspective-taking, and practice emotion talk in a safe and meaningful way (Mar et al., 2011; Oatley, 2016; Schapira and Aram, 2020). Furthermore, SBR is uniquely suited to facilitate adult-child emotion dialogue and socialization processes in early years, making it a powerful vehicle for fostering social and emotional competence through existing pedagogical practices.

In this paper, we first introduce the practice of SBR, define social and emotional competence, and outline the main modes of emotion socialization. We then review articles reporting on interventions based on SBR and emotion discourse that aimed to enhance children’s social and emotional competence. Finally, we discuss the key implications of the narrative review, highlighting the initial achievements of research in this area and identifying the current gaps that need to be addressed in future work, both in research and in the work of educators in early childhood settings to promote preschool’s and toddler’s social and emotional competences.

## 2 Shared book reading

Vygotsky’s (1978) sociocultural theory posits that young children’s learning is inherently dyadic, occurring through social interactions with a more knowledgeable individual (e.g., a parent or teacher). Children’s development is embedded in social settings and practices. Shared book reading (SBR) is a daily activity that exemplifies this social learning process, providing a natural and dynamic setting for meaningful adult-child discourse. SBR plays a crucial role in fostering child development across multiple domains, including language, social interaction, and cognitive growth (e.g., Mol et al., 2008). More than just the frequency of reading, the quality of interaction during SBR is essential, as it encourages children to engage in conversation, articulate thoughts, expand their vocabulary, and develop narrative skills—all fundamental to cognitive and linguistic growth. One particularly effective approach is Dialogic Book-Sharing (DBS), which actively engages children in conversation, questioning, and storytelling (Bus et al., 1995; Hargrave and Sénéchal, 2000; Ornaghi et al., 2024; Whitehurst et al., 1988). Effective DBS practices, supported by specific adult behaviors such as gaze-following, can enhance toddlers’ cognitive development and contribute to their educational success (Murray et al., 2022). For example, a meta-analysis by Dowdall et al. (2020) examined shared picture book reading interventions across 19 studies (*N* = 2,594, ages 1–6), finding positive effects on expressive (*d* = 0.41) and receptive (*d* = 0.26) language. The analysis showed that higher-intensity interventions are more effective, highlighting the importance of structured and interactive reading techniques. In addition, interventions that teach educators to expand dialogue and conduct interactive book readings make a unique contribution to children’s language development (e.g., Rezzonico et al., 2015; Wasik and Hindman, 2020) and inferential comprehension (e.g., Grolig et al., 2020).

SBR is a common practice in early childhood classrooms, conducted in whole-class or small-group settings. Studies have identified key adult behaviors that enhance children’s learning during SBR, including reading aloud, actively engaging children, providing positive reinforcement, and encouraging higher-order thinking (Batini et al., 2020; Schmidt et al., 2024; Wasik and Bond, 2001; Whitehurst et al., 1994). These behaviors foster an interactive and collaborative experience for children. Teachers facilitate active participation by asking questions, scaffolding, encouraging predictions, and engaging students in discussions about the text (Deshmukh et al., 2019; Zucker et al., 2020). Recent research has increasingly examined factors influencing the quality of shared reading interactions, including the characteristics of the books themselves (e.g., Bojczyk et al., 2016). The key components of a book—its illustrations, language, socio-emotional themes, and narrative structure—play a crucial role in shaping shared reading experiences and their outcomes (e.g., Garner et al., 2008; Hoffman et al., 2015).

Books come in a variety of genres, including narrative stories, poems, and informational texts, each influencing the nature of shared reading interactions. Narrative stories typically present a structured sequence of events involving characters, poetic texts emphasize rhythm and language play, supporting both affective and linguistic engagement, and plot informational texts provide factual content and are often used to explain concepts or phenomena (Barone, 2010; Larson, 2015) given that adults and children engage differently depending on the book’s genres (e.g., Bergman Deitcher et al., 2019; Schapira et al., 2021). For instance, reading informational texts tends to prompt more cognitively complex discussions between adults and children (e.g., Price et al., 2009). Additionally, Moschovaki and Meadows (2005) found that integrating a familiar narrative style into an informational book elicited more predictive and analytical responses from children.

SBR engages three essential components: the adult, the child, and the book. The studies we have mentioned highlight the contributions of dialogue and interaction during SBR to young children’s language development and narrative skills (e.g., Mol et al., 2008). In recent years, however, there has been growing recognition of the unique role that SBR can play in young children’s social–emotional development (e.g., Aram et al., 2017; Schapira and Aram, 2020). In this study, we specifically examine how SBR interventions in educational settings contribute to enhancing children’s emotional competence.

## 3 Social and emotional competence and its socialization

Emotional or social–emotional competence (SEC) can be defined as the ability to effectively navigate emotion-eliciting encounters (Saarni, 2008; Denham, 2023). It includes (1) experiencing and purposefully expressing a broad variety of emotions, considering parameters such as duration and intensity; (2) regulating emotions in accordance with one’s own comfort and the needs of others; and (3) understanding both one’s own and others’ emotions, particularly in relation to their underlying causes. Emotion expressiveness, knowledge, and regulation encompass a variety of abilities that develop over time. The period from 18 months to approximately 5 or 6 years, covering toddlerhood and the preschool period, represents a crucial phase of development, when children rapidly advance in motor, language, cognitive, and social–emotional competence. Within the domain of social and emotional development in particular, the socialization of emotional competence has been extensively studied (for a narrative review: Grazzani and Conte, 2024). This process involves three key emotion socialization practices used by adults in daily interactions with children: modeling, contingency, and teaching. Modeling refers to the way children observe the emotions expressed by their socializers and assimilate their expressive behaviors. By watching their parents, children learn a great deal about emotions and feelings—for example, how to label emotions and associate them with specific types of events. Contingency refers to socializers’ reactions to children’s emotions, which encompass a variety of actions, including behavioral and emotional encouragement or discouragement (Denham, 2007). Positive reactions, such as tolerance, comfort, acceptance, convey the message that emotions can be managed and are even beneficial (Gottman et al., 1997). In contrast, negative reactions suggest that emotions are problematic and should be repressed. Teaching practice refers to socializers’ tendency to discuss emotions within a warm and supportive relationship. It involves guiding children in expressing and regulating their emotions, using emotion talk to help them interpret their experiences and behavior. Many studies (for a review: Denham, 2023) suggest that adult-child conversations about feelings and emotions provide a crucial context for coaching children in understanding both their own and others’ emotions.

Emotion socialization can occur both during parent–child interactions and in extra-familial settings. A substantial body of empirical evidence exists on the parental socialization of emotions, given that parents serve as children’s primary socializers. They play a key role in early processes of emotion co-regulation (Trevarthen, 1993), helping infants gradually learn to manage their emotions independently. This process continues throughout infancy and toddlerhood, becoming enriched by numerous additional interactions and relationships, including those with siblings and peers (Reddy, 2008).

Compared to the numerous studies conducted in family contexts, the educational environment has received less attention, even though educators and teachers play a crucial role as socializers of emotional competence (Denham, 2023). Research on educators’ and teachers’ beliefs about the role of emotions reveals a variety of perspectives. For example, beliefs differ regarding the extent to which teachers should help children to regulate their emotions or teach them the rules for displaying emotion (Ahn, 2005).

Given that the development of emotional competence is linked to various outcomes such as social competence, popularity among peers, prosocial behavior, mental health, and academic achievement (Denham, 2007), early intervention to enhance emotional competence in childhood serves as a key protective factor in children’s developmental trajectories. Hence, this narrative review concentrates on studies that have documented the impact of a specific mode of teaching or coaching for the socialization of emotions—namely, SBR followed by group discussions on emotions within the community life context of nurseries and kindergartens. The tendency to discuss emotions, and to use them to clarify, teach, or share, rather than to modify children’s behavior, helps children express and regulate their own emotions, thereby advancing their emotional competence.

## 4 SBR and social and emotional competence

According to the coaching approach within the emotion socialization perspective, adults can engage children in discussions about emotions during SBR. Mar et al. (2009) and Oatley (2016) suggested that reading narrative fiction shapes our understanding of the social world by allowing readers to mentally simulate a broad spectrum of emotions and social experiences. Children’s books, in particular, frequently portray interactions among humans, animals, or anthropomorphized creatures, providing opportunities to explore story characters’ emotions, thoughts, intentions, beliefs, and desires. These narratives naturally foster discussion about social and emotional experiences, helping children navigate complex social situations (Cassidy et al., 1998).

During shared book reading, discussions often extend beyond the basic storyline to include both the “explicit” aspects of the book—such as the plot, illustrations, and characters (Price et al., 2009)—and the “implicit” aspects that demand deeper cognitive and emotional engagement. These implicit elements encourage children to reflect on social and emotional themes, make connections to their own experiences, and practice perspective-taking (Hindman et al., 2014). Thus, shared reading promotes critical thinking and enhances children’s socio-emotional competence. This concept aligns with Bruner’s (1986, 1990) dual landscape model, which posits that narratives comprise two interwoven “landscapes.” The landscape of action relates to the observable elements of the story, including the sequence of events and characters’ behaviors. Conversely, the landscape of consciousness delves into the narrative’s underlying psychological and emotional dimensions, including the thoughts, beliefs, desires, emotions, and intentions that drive characters’ actions. Engaging children in both the landscapes of action and consciousness during shared reading facilitates richer, more meaningful interactions with the story, fostering deeper comprehension and emotional engagement. Bassett et al. (2020) found that preschool teachers’ discussions about emotions during SBR were particularly beneficial for children with more limited emotion knowledge. However, there were noticeable differences among educators in terms of their use of emotional language and book-reading styles in preschool settings. Similarly, Misailidi et al. (2013) observed variability in kindergarten teachers’ use of mental state language during storytelling, underscoring differences in how educators integrate discussions about thoughts, feelings, and desires into their narratives.

These findings underscore the importance of considering the triadic nature of shared book reading—the adult, the child, and the book—as an interactive system. The adult guides the process, scaffolding the child’s understanding and engagement; the child brings their developmental capacities, curiosity, and emotional experiences; and the book serves as both a narrative and emotional anchor. The interplay between these components is especially salient in fostering emotional discourse and perspective-taking, thereby enhancing socio-emotional competence (Schapira and Aram, 2020).

In conclusion, intervention programs provide educators with opportunities to develop and refine their ability to engage emotional discourse, ultimately supporting children’s development of SEC within educational settings. While most studies and interventions on SBR and emotional competence have focused on family settings, this narrative review examines the impact of SBR interventions in nursery and preschool contexts, emphasizing the critical role of these educational settings in enhancing children’s social and emotional skills.

## 5 Methodological approach: Narrative review

Leveraging our own prior expertise in the research field of shared book reading (SBR) in educational contexts, we conducted a comprehensive literature search using the narrative review methodology (e.g., Sukhera, 2022) across the main platforms for academic literature, namely PubMed, Google Scholar, Scopus, and Web of Science. Narrative reviews are particularly useful for synthesizing diverse evidence and identifying patterns, gaps, and emerging themes across studies that use different methodologies and approaches. Given the exploratory nature of this review, the narrative approach allowed us to incorporate findings from a variety of contexts, including nursery and preschool settings, and to provide a holistic understanding of how SBR interventions influence children’s social and emotional competence. To guide our search, we applied a range of targeted keywords, such as SBR in nursery contexts, SBR in preschool/kindergarten settings, SBR and social and emotional competence, SBR and conversation on emotions, and SBR and emotion discourse/language. The inclusion criteria were as follows: (i) empirical studies examining the implementation of SBR-based interventions or programs, (ii) empirical studies conducted in educational settings, and (iii) studies focusing on children aged between 1.5 and 6 years. Studies conducted in primary school settings were excluded from this narrative review to maintain the focus on early childhood education. The initial search results were screened for duplicates and reviewed by both authors, who independently assessed titles, abstracts, and full texts to determine eligibility. Any disagreements were resolved through discussion and consensus.

Applying these criteria, we identified 15 empirical studies: 5 conducted in nursery settings and 10 in preschool environments. The selected studies (see Table 1) focused on SBR-based interventions and emotion-focused discourse in educational contexts, aiming to enhance various aspects of children’s social and emotional competence. Adopting a narrative review approach, we organized and interpreted the studies, highlighting promising practices as well as gaps that warrant further exploration.

**Table 1:** Characteristics of the intervention studies listed alphabetically by Author.

Authors	Educational setting	Trainer	Number and age of participants	Duration of intervention	Target components of SEC	Measurement tools
Bergman Deitcher et al. (2021)	Preschool	M.A. students	116 preschoolers (*M* = 75; SD = 0.28)	30-min sessions for 2 weeks	Emotion lexicon and social problem-solving skills	Direct measures
Betawi (2015)	Nursery	Teacher	10 toddlers (24–36 months)	Daily sessions lasting 5–8 min for 3 weeks	Social and emotional skills (in general)	Indirect measure
Brazzelli et al. (2021)	Nursery	Teachers	142 toddlers (*M* = 28.5 months; SD = 3.92)	2 months	Prosocial behaviors and empathy	Direct measures (validated tasks) and indirect measure (validated parent questionnaire)
Giménez-Dasí et al. (2013)	Preschool	Teachers	60 preschoolers (4 years old: *M* = 52 months; 5 years old: *M* = 64 months)	30 weekly sessions	Emotion comprehension and strategies for social interaction	Direct measures (validated tests)
Giménez-Dasí et al. (2017)	Preschool	Two trained teachers per class	43 preschoolers (*M* = 59.8 months)	22 weekly sessions	Emotion comprehension and strategies for social interaction	Direct measures (validated tests)
Grazzani and Ornaghi (2011)	Preschool	Researcher	100 preschoolers (3–5 years; *M* = 52 months; SD = 9.9)	Two-month period (using 16 stories developed by the authors)	Emotion comprehension	Direct measures (validated tests)
Grazzani et al. (2016a)	Nursery	Teachers	105 toddlers 21–36 months (*M* = 29.8; SD = 3.78)	Two-month period: at least four sessions a week using eight stories developed *ad hoc*	Emotion talk, emotion comprehension, and prosocial behavior	Direct measures (validated tasks), video observations and indirect measure (validated parent questionnaire)
Grazzani et al. (2016b)	Nursery	Teachers	68 toddlers (24–35 months; M = 29.9 months; SD = 3.50)	Daily 10-15-min training sessions for 1 month; stories developed by the authors	Emotion comprehension and emotional theory of mind	Direct measures (validated tasks) and indirect measure (parent questionnaire)
Grazzani and Valle (2025)	Preschool	Researchers	61 preschoolers (3–6 years; *M* = 54 months; SD = 9.8)	Two-month period: twice-weekly training sessions using eight stories developed ad hoc	Emotion understanding and prosocial orientation	Direct measures (validated tasks)
Ornaghi et al. (2017)	Nursery	Teachers	95 toddlers 26–36 months (*M* = 29.8 months; SD = 3.78)	42 × 15-min intervention sessions over 2 months	Emotion comprehension and prosocial behavior	Direct measures (validated tasks), indirect measure (validated parent questionnaire) and video observations
Ornaghi et al. (2011)	Preschool	Researcher	70 preschoolers 35–57 months (*M* = 3.10 months; SD = 5.96)	Twice-weekly 20-min sessions over 2 months, based on 16 stories developed by the authors	Emotion comprehension and comprehension of emotion verbs	Direct measures (validated tasks)
Ornaghi et al. (2015)	Preschool	Researcher	75 preschoolers (*M* = 60.1 months; SD = 6.83)	12 sessions conducted twice weekly over 6 weeks	Emotion comprehension and prosocial orientation	Direct measures (validated tasks)
Schapira (2024)	Preschool	Pre-service teacher	77 preschoolers (*M* = 54.95 months; SD = 23.19)	One session per week for 6 weeks	Empathy	Direct measures (validated tests)
Schapira et al. (2024)	Preschool	Pre-service teacher	205 preschoolers (*M* = 56.60 months; SD = 10.34)	One session per week for 6 weeks	Empathy and social values	Direct measures (validated tests)
Voltmer and von Salisch (2022)	Preschool	Teachers	110 preschoolers 36–66 months (*M* = 49.41 months; SD = 7.23)	5 months, with six modules completed in 40 h	Emotion knowledge	Direct measures (validated tests)

## 6 Intervention studies in nursery and preschool contexts

In what follows, we describe the empirical studies identified in this narrative review, focusing on SBR-based interventions conducted in nursery and preschool settings. Each study is presented with attention to its design, population, intervention format, and key findings related to the promotion of children’s emotional and social competences. With regard to SBR in nursery environments, Betawi (2015) conducted one of the earliest studies focusing on shared story reading and its effects on toddlers’ social and emotional competence. The research design included a single intervention group (*N* = 10), who participated in daily training sessions lasting approximately 5–8 min, without a control group for comparison. The intervention began with the teacher presenting the cover of a book from a selected collection. She then read the title and introduced the story to elicit children’s prior knowledge about the story theme and build background understanding. The teacher engaged the children with questions such as: What do you see on the cover of the book? What do you think the story is about? Do you have a dog at home? What is its name? What is the girl doing? What can you do? Can you behave like that? Would you like to do that? The teacher was asked to evaluate each child twice via a questionnaire, completed both before introducing the stories and at the intervention’s conclusion. Betawi (2015) reported significant improvements between pre and post-test on items such as “the child enjoys sharing laughter with others,” “the child says thank you when appropriate,” and “the child shares with others.” The main limitations of this potentially insightful study, which emphasizes story reading and discussion to enhance children’s emotional competence, include the lack of a control group, a small sample size, the use of a non-validated questionnaire, and the absence of direct measures.

In a study by Grazzani et al. (2016a), a quasi-experimental design was used to evaluate the effectiveness of an intervention based on SBR and discussions about emotions with small groups of 2- to 3-year-old children. Participants were 105 toddlers ranging from 21 to 36 months at the beginning of the study. The children assigned to the experimental group took part in training sessions conducted in small groups (4/6 children per group) over a two-month period. Each child participated in the sessions at least four times a week throughout the intervention phase. The stories, specifically developed for the study, were presented in a book format. The main characters, two rabbits, experience a range of emotions—fear, happiness, anger, sadness—through a series of adventures. The narratives adhered to a traditional story schema: after the scene has been set, a critical situation eliciting a particular emotion arises, and action is required to resolve the ensuing crisis. The story texts were enriched with emotional language (e.g., gets mad, is scared, is surprised, is happy, and so on), and other mental state terms. The training sessions with the experimental group followed a four-step procedure: setting the appropriate context for the activity, reading the story, discussing the emotion thematized in the story, and concluding with a windup stage. The crucial element of this procedure was the conversation about emotions, which was conducted with small groups of young children. In contrast, the control group only participated in the first two steps. The authors reported that the training group significantly outperformed the control group in both the use of emotional state lexicon and in understanding emotion.

Further analysis of this data corpus by the researchers revealed additional findings regarding a sub-sample of 95 toddlers aged 26- to 36-month-old (Ornaghi et al., 2017). The toddlers who participated in the intervention described above, based on SBR and conversations about emotions, displayed a significantly higher propensity to engage in prosocial behaviors compared to the control group, who listened to the stories but did not participate in conversations about emotions.

Brazzelli et al. (2021) conducted an intervention study where trained teachers read prosocial stories to small groups of toddlers and facilitated conversations about the featured emotions, following specific guidelines. The sample comprised 142 toddlers, divided into three groups/conditions, who participated in a two-month program conducted three times a week. In condition 1, toddlers participated in SBR followed by conversations on emotions and other inner states (TEPP: *Toddlers Empathy Prosociality Program*). In condition 2, children participated in SBR and conversation about concrete actions and physical states related to the story characters. In condition 3, after listening to the stories, toddlers engaged in free play without structured conversations with adults. The authors observed that toddlers in condition 1 significantly improved both their empathy and prosocial behaviors compared to the other groups, concluding that SBR and conversation on emotions can make a key contribution to enhancing toddlers’ socio-emotional skills. These findings align with those of a smaller study by Grazzani et al. (2016b), in which 68 toddlers were divided into an experimental group (33 toddlers participating in shared story reading and conversation about emotions) and a control group (35 toddlers engaging in shared story reading and conversation about material entities and actions featured in the storybook). The experimental group significantly outperformed the control group on measures of emotion understanding.

Let us now turn to SBR studies conducted in preschool settings. Ornaghi et al. (2011) carried out a study with 70, 3- to 4-year-old children randomly assigned either an experimental or a control group. During a two-month intervention, children in small groups attended SBR sessions, where they listened to 16 stories enriched with mental state language, including both emotional and cognitive lexicon. Following a structured program developed by the authors, the experimental group took part in *language games* and conversations designed to encourage the use of mental terms after each story reading session. In contrast, the control group did not participate in any structured or targeted conversational activities. Following the intervention, the experimental group outperformed the control group on both language and emotion comprehension measures.

Utilizing the same SBR-based program, which included the *word launch technique* and *language games*, and focusing specifically on stories that highlighted emotion terms, Grazzani and Ornaghi (2011) achieved comparable outcomes with children aged 3–5 years (range: 35–70 months). The conversations about emotions, conducted in strict fulfilment of the program guidelines (for a recent detailed description of the validated program, see Grazzani and Ornaghi, 2022), significantly improved emotion comprehension, particularly among 4-year-old participants. In a recent study, Grazzani and Valle (2025) used this same program, that they called PSULG (Promoting Social Understanding through Language Games), with a new sample of 61 preschoolers (range: 38–71 months). Controlling for age, the authors found that the training had a significant impact on participants’ emotion understanding and a marginally significant effect on their prosocial orientation.

In a study by Ornaghi et al. (2015), a six-week intervention was implemented in a preschool setting. Children, in small groups, were presented with brief illustrated stories based on emotional scripts. Following the shared story reading, the training group (*N* = 38) engaged in discussions about the nature, causes, and regulation of emotions, while the control group (*N* = 35) participated in free play. The authors reported that the intervention, which included both SBR and conversations about emotions, significantly enhanced emotion comprehension and prosocial orientation in the experimental group compared to the control group. Furthermore, they noted that the positive effects of the intervention remained stable 4 weeks after the project concluded.

Giménez-Dasí et al. (2013) examined the impact of a *Philosophy for Children: Thinking Emotions* intervention on the emotional and social development of 60 preschool children (4- and 5-year-olds) from middle-class families in Madrid. The study involved four preschool classes, with all children participating; two classes served as experimental groups and two as control groups. The year-long intervention consisted of 30 weekly sessions (1 h each), following a three-step approach: story reading, guided discussion, and interactive activities (e.g., drawing, role-playing). Teachers facilitated discussions without providing answers and concluded each session with a summary of key ideas. The program included 16 sessions on reading, questioning, and dialogue, 10 sessions on specific emotional topics, and 6 sessions on coping strategies to help children manage negative emotions and develop prosocial behavior. The parents of the experimental group children were involved in complementary activities at home once a month, while prior to the intervention the teachers received training in facilitating discussions and delivering emotional coaching. The authors found that the 5-year-olds in the intervention group displayed gains in social performance and emotion knowledge, whereas the 4-year-olds only displayed increased competence in navigating social interactions without a corresponding improvement in explicit emotion knowledge. These findings underscore the effectiveness of Philosophy for Children (P4C) interventions and the need for age-specific adaptations to optimally enhance children’s social and emotional skills.

Another study by Giménez-Dasí et al. (2017) used the *Philosophy for Children: Thinking Emotions* program to enhance emotion knowledge and social interaction strategies with 43 Roma children (aged 4–5) in Valencia, Spain. Roma children, one of the largest minority groups in Spain and Europe, often live in poverty, face social exclusion, and present delays in cognitive, linguistic, and emotional abilities. The children were divided into experimental (*N* = 21) and control (*N* = 22) groups, with the 22-session intervention led by two trained teachers per class. The experimental group displayed significant improvements, particularly in emotion knowledge and social interaction strategies.

A further study in a low socio-economic status (SES) population by Bergman Deitcher et al. (2021) examined the impact of a small-group SBR intervention on mental–emotional conceptualization and social understanding among 116 Arab preschoolers, aged 68–80 months (65 girls) from 13 kindergartens in northern Israel. The study used the wordless picture book “When Night Fell” with two Arabic text versions: one focusing on mental–emotional states (intervention) and the other on character descriptions and actions (comparison). Children were randomly assigned to intervention or comparison groups. They participated in five 30-min sessions over a two-week period, conducted by trained M.A. students. The authors reported that the intervention group displayed significantly greater gains in their mental-emotion lexicon and social problem-solving skills.

Voltmer and von Salisch (2022) developed the *Feeling Thinking Talking (FTT)* intervention to train early childhood educators in language support strategies (LSS) for enhancing language skills and emotion knowledge in young children. Conducted at five early childhood centers in a low-income district of Hamburg, Germany, the study involved 110 children (*M* = 49.41 months), divided into an FTT intervention group (*N* = 43) and a “business-as-usual” (BAU) control group (*N* = 67). The intervention included a 40-h teacher training program covering six modules, focused on the application of LSS techniques during SBR, reminiscing, and everyday conversations. The FTT intervention was implemented over a five-month period. The children in the FTT group displayed greater progress in emotion knowledge, particularly in understanding mixed emotions, with significant effects persisting at follow-up. Two studies presented at international conferences and published in peer-accessible conference proceedings explored the impact of SBR interventions on preschoolers’ empathy and social values (Schapira, 2024; Schapira et al., 2024). The first study examined how SBR intervention programs contribute to preschoolers’ empathy. A first study focused on the emotional discourse of pre-service educators on teaching practice in preschools, particularly while interacting with small groups of preschoolers during SBR sessions using both narrative and educational texts. Participants were 20 pre-service preschool teachers and 77 preschoolers (*M* = 54.95 months, 34 boys, 43 girls). The pre-service educators were first trained in emotional discourse and then randomly assigned to an intervention or a control group. The intervention group participated in six SBR sessions enriched with emotion discourse, while the control group participated in six book-reading sessions without conversation. Both groups displayed significant overall gains in empathy, with boys initially scoring lower than girls but showing greater gains post-intervention. These outcomes suggest that the emotion discourse strategies learned by the pre-service teachers before the intervention program were effectively applied to various situations in the preschool setting, enhancing children’s empathy in both intervention and control groups. The second study (Schapira et al., 2024) investigated empathy and social values (children’s perspective on *universality*, *benevolence*, and *power*) in preschoolers. Twenty pre-service preschool teachers, on teaching practice in preschools, worked with 205 children (*M* = 56.60 months). Each teacher randomly assigned four children to an intervention group and four to a control group. The intervention group participated in six SBR sessions that included guided discourse on emotions, while the control group read the same books without any discussion. The book selected for the intervention focused on themes of cooperation, helping others, and promoting goodness. Relative to the control group, the intervention group displayed significantly enhanced empathy, but no significant changes in social values. This study was the first to examine the link between empathy and social values in young children, complementing existing research conducted with adolescents and adults. Table 1 summarizes the main characteristics of all the reviewed studies.

## 7 Conclusion

This narrative review underscores the potential to leverage SBR as an effective educational tool for enhancing social and emotional competence in early childhood, a critical period for the development of these abilities (Denham, 2007; Harris et al., 2016). Although these interventions are time-consuming, they highlight the importance of starting early to use books and engage in emotion discourse to promote children’s social and emotional skills. However, few studies have likely explored this approach extensively due to the considerable time and investment required to implement such program. Given that children spend a significant portion of their day in educational settings, nurseries and preschools offer ideal environments for fostering emotional learning and development (Denham, 2023). The studies discussed in this review affirm the view that teachers play a crucial role as emotion socializers for young toddlers and preschoolers, specifically because they have privileged opportunities to engage in emotion discourse with children.

In this conclusion, we highlight key considerations aimed at both enhancing the practical implementation of SBR in educational contexts and informing future research on this topic.

### 7.1 Training educators and teachers

It is essential to equip educators and teachers not only with practical competence in SBR but also with a thorough understanding of social and emotional competence research (Ferreira et al., 2025). We cannot assume that education practitioners are familiar with this area of scientific inquiry. Training in the practices of shared book reading and conversation about emotions provides teachers with a powerful tool for fostering children’s emotional competence. Through guided discussions with a teacher, children progressively learn to identify the emotions of story characters, recognize their own emotions, differentiate their feelings from those of others, regulate their emotions, and even develop empathy across a variety of scenarios.

### 7.2 Choice of book

Most of the studies reviewed used books or stories specifically written for their respective research or intervention programs. However, three studies utilized commercially available story books. It is crucial to continue exploring a diverse range of books across various genres to evaluate their effectiveness in fostering emotional discourse. Selecting books grounded in pedagogical and psychological principles enables practitioners to implement high-quality activities centered on discussing emotions while focusing on the landscapes of both action and consciousness within the stories. Books that feature texts and illustrations dedicated to the inner world of emotions provide an excellent starting point for discussion. Such conversations can delve into not only the explicit aspects of the stories, such as the actions of the protagonists, but also the implicit, internal dimensions of the story characters and the children themselves.

### 7.3 Shared book reading in small groups

Most of the interventions reviewed [except the studies by Giménez-Dasí et al. (2013, 2017) and by Voltmer and von Salisch (2022)] were conducted with small groups, facilitating meaningful interactions between adult and children. In these settings, guided discussions during SBR sessions help the children *as a group* not only to develop an understanding of emotions and refine their ability to regulate feelings, but also to enhance their perspective-taking skills. Educators and teachers play a critical role in these discussions, helping the children in the group to actively engage in conversation about emotions. This direct involvement helps children to distinguish and compare their own emotions and those of others, fostering a greater awareness of others’ perspectives and feelings. This interaction can be understood through the essential triadic structure of SBR: the adult, the child, and the book. The adult mediates between the book’s narrative content and the child’s internal world, enabling personal connection and emotional reflection. The child, in turn, actively contributes by making sense of the story and relating it to their own experiences, while the book provides a structured context that evokes discussion and learning (Mar et al., 2011; Mol et al., 2008). Recognizing this dynamic interplay can guide the development of more nuanced intervention programs.

With regard to future research, it would be useful to include a follow-up phase in intervention studies to evaluate the long-term effects of SBR and discussion of emotions. Currently, only two studies in the present narrative review reported follow-up findings (Ornaghi et al., 2015; Voltmer and von Salisch, 2022). Additionally, exploring the impact of these interventions on diverse populations, such as children from low socioeconomic backgrounds or those with special needs, could provide valuable insights. In fact, there is limited research demonstrating significant benefits of SBR interventions for children in disadvantaged settings, yet these findings suggest that structured discussions on emotions can effectively support social and emotional development in challenging contexts (Bergman Deitcher et al., 2021; Giménez-Dasí et al., 2017; Voltmer and von Salisch, 2022).

Building on the initial promising results from studies conducted in the nursery context, we believe that further research should focus specifically on toddlerhood. Numerous studies have shown that social emotional competence begins to develop from the first year of life (Trevarthen, 1993) and that even very young children can actively participate in social interaction, thereby learning about the social and emotional world. As the groundwork for emotional understanding and social competence is established during these formative years, it becomes increasingly important to introduce SBR and emotion discourse early to ensure the greatest developmental impact. Thus, future research could delve deeper into optimizing the characteristics of SBR and conversations about emotions. This could include exploring the optimal duration of sessions, determining the most effective number of participants in group activities, and identifying the best methods for scaffolding toddlers’ learning through questions, comments, and other forms of dialogue.

Additionally, emotional socialization practices are shaped by cultural values and norms, influencing how adults talk about emotions with children (Basabe et al., 2000; Kılıç et al., 2025). For example, Carmiol and Schröder (2019) found that Costa Rican mothers engaged in more frequent emotion talk during SBR compared to German mothers. Despite the growing interest in SBR interventions, little is known about how cultural factors affect the way teachers engage in emotion discourse during reading. Future research should therefore explore cross-cultural differences in SBR practices to better understand emotional socialization processes and to inform the design of culturally sensitive interventions that consider the values, communication styles, and emotional practices of diverse populations.

Overall, SBR combined with structured emotion discourse serves as a powerful mechanism for fostering social and emotional competence among young children in educational settings. By embedding emotion discourse into early childhood education, educators can significantly enhance children’s social and emotional abilities. These abilities are crucial because they are correlated with positive outcomes such as wellbeing, academic achievement, popularity among peers, and a predisposition towards positive social behaviors (Denham, 2023).

## References

[ref1] Ahn H. J. (2005). Child care teachers’ beliefs and practices regarding socialization of emotion in young children. J. Early Child. Teach. Educ. 26, 283–295. doi: 10.1080/10901020500371155

[ref2] Aram D. Deitcher D. B. Shoshan T. S. Ziv M. (2017). Shared book reading interactions within families from low socioeconomic backgrounds and children’s social understanding and prosocial behavior. J. Cogn. Educ. Psychol. 16, 157–177. doi: 10.1891/1945-8959.16.2.157

[ref3] Barone D. M. (2010). Children’s literature in the classroom: engaging lifelong readers. New York, NY: Guilford Press.

[ref4] Basabe N. Paez D. Valencia J. Rimé B. Pennebaker J. Diener E.. (2000). Sociocultural factors predicting subjective experience of emotion: a collective level analysis. Psicothema 12, 55–69.

[ref5] Bassett H. H. Denham S. A. Mohtasham M. Austin N. (2020). Psychometric properties of the book readings for an affective classroom education (BRACE) coding system. Read. Psychol. 41, 322–346. doi: 10.1080/02702711.2020.1768980

[ref6] Batini F. D’Autilia B. Pera E. Lucchetti L. Toti G. (2020). Reading aloud and first language development: a systematic review. J. Educ. Train. Stud. 8, 49–68. doi: 10.11114/jets.v8i12.5047

[ref7] Bergman Deitcher D. Aram D. Khalaily-Shahadi M. Dwairy M. (2021). Promoting preschoolers’ mental-emotional conceptualization and social understanding: a shared book-reading study. Early Educ. Dev. 32, 501–515. doi: 10.1080/10409289.2020.1772662

[ref8] Bergman Deitcher D. Johnson H. Aram D. (2019). Does book genre matter? Boys’ and girls’ word learning from narrative and informational books in the preschool years. J. Res. Read. 42, 193–211. doi: 10.1111/1467-9817.12266

[ref9] Betawi I. A. (2015). What effect does story time have on toddlers’ social and emotional skills. Early Child Dev. Care 185, 594–600. doi: 10.1080/03004430.2014.943756

[ref10] Bojczyk K. E. Davis A. E. Rana V. (2016). Mother–child interaction quality in shared book reading: relation to child vocabulary and readiness to read. Early Child Res. Q. 36, 404–414. doi: 10.1016/j.ecresq.2016.01.006

[ref11] Brazzelli E. Grazzani I. Pepe A. (2021). Promoting prosocial behavior in toddlerhood: a conversation-based intervention at nursery. J. Exp. Child Psychol. 204:105056. doi: 10.1016/j.jecp.2020.105056, PMID: 33341017

[ref12] Bruner J. (1986). Actual minds, possible worlds. Cambridge, MA: Harvard University Press.

[ref13] Bruner J. S. (1990). Act of meaning. Cambridge, MA: Harvard University Press.

[ref14] Bus A. G. Van Ijzendoorn M. H. Pellegrini A. D. (1995). Joint book reading makes for success in learning to read: a meta-analysis on intergenerational transmission of literacy. Rev. Educ. Res. 65, 1–21. doi: 10.3102/00346543065001001

[ref15] Carmiol A. M. Schröder L. (2019). Emotion talk during mother–child reminiscing and book sharing and children’s socioemotional competence: evidence from Costa Rica and Germany. Cult. Brain 7, 126–147. doi: 10.1007/s40167-019-00078-x

[ref16] Cassidy K. W. Ball L. V. Rourke M. T. Werner R. S. Feeny N. Chu J. Y.. (1998). Theory of mind concepts in children's literature. Appl. Psycholinguist. 19, 463–470. doi: 10.1017/S0142716400010274

[ref17] Denham S. A. (2007). Dealing with feelings: how children negotiate the worlds of emotions and social relationship. Cogn. Brain Behav. 11, 1–48. https://denhamlab.gmu.edu/Publications%20PDFs/Denham%202007.pdf

[ref18] Denham S. A. (2023). The development of emotional competence in young children. New York, NY: Guilford Press.

[ref20] Deshmukh R. S. Zucker T. A. Tambyraja S. R. Pentimonti J. M. Bowles R. P. Justice L. M. (2019). Teachers’ use of questions during shared book reading: relations to child responses. Early Child Res. Q. 49, 59–68. doi: 10.1016/j.ecresq.2019.05.006

[ref21] Dowdall N. Melendez-Torres G. J. Murray L. Gardner F. Hartford L. Cooper P. J. (2020). Shared picture book reading interventions for child language development: a systematic review and meta-analysis. Child Dev. 91, e383–e399. doi: 10.1111/cdev.13225, PMID: 30737957

[ref22] Ferreira M. Reis-Jorge J. Olcina-Sempere G. (2025). Social and emotional learning—Portuguese and Spanish teachers’ representations and classroom practices. Front. Educ. 9:1461964. doi: 10.3389/feduc.2024.1461964

[ref23] Garner P. W. Dunsmore J. C. Southam-Gerrow M. (2008). Mother–child conversations about emotions: linkages to child aggression and prosocial behavior. Soc. Dev. 17, 259–277. doi: 10.1111/j.1467-9507.2007.00424.x

[ref24] Giménez-Dasí M. Quintanilla L. Daniel M. F. (2013). Improving emotion comprehension and social skills in early childhood through philosophy for children. Child. Philos. 9, 63–89.

[ref25] Giménez-Dasí M. Quintanilla L. Ojeda V. Lucas-Molina B. (2017). Effects of a dialogue-based program to improve emotion knowledge in Spanish Roma preschoolers. Infants Young Child. 30, 3–16. doi: 10.1097/IYC.0000000000000086

[ref26] Gottman J. M. Katz L. F. Hooven C. (1997). Meta-emotion. How families communicate emotionally. Mahwah, NJ: Erlbaum.

[ref27] Grazzani I. Conte E. (2024). Lo sviluppo della comprensione sociale. Una rassegna narrativa focalizzata sul linguaggio nella fascia 0-6 anni. Psicol. Clin. dello Sviluppo 28, 29–58. doi: 10.1449/112819

[ref28] Grazzani I. Ornaghi V. (2011). Emotional state talk and emotion understanding: a training study with preschool children. J. Child Lang. 38, 1124–1139. doi: 10.1017/S0305000910000772, PMID: 21426620

[ref29] Grazzani I. Ornaghi V. (2022). Children’s development of social understanding. Parma: Junior.

[ref30] Grazzani I. Ornaghi V. Agliati A. Brazzelli E. (2016a). How to foster toddlers’ mental-state talk, emotion understanding, and prosocial behavior: a conversation-based intervention at nursery school. Infancy 21, 199–227. doi: 10.1111/infa.12107

[ref31] Grazzani I. Ornaghi V. Brockmeier J. (2016b). Conversation on mental states at nursery: promoting social cognition in early childhood. Eur. J. Dev. Psychol. 13, 563–581. doi: 10.1080/17405629.2015.1127803

[ref32] Grazzani I. Valle M. (2025). Training preschoolers with language games: enhancing theory of mind, emotion understanding, prosocial orientation, and language ability. Eur. J. Dev. Psychol., 1–23. doi: 10.1080/17405629.2025.2523752

[ref33] Grolig L. Cohrdes C. Tiffin-Richards S. P. Schroeder S. (2020). Narrative dialogic reading with wordless picture books: a cluster-randomized intervention study. Early Child Res. Q. 51, 191–203. doi: 10.1016/j.ecresq.2019.11.002

[ref34] Hargrave A. C. Sénéchal M. (2000). A book reading intervention with preschool children who have limited vocabularies: the benefits of regular reading and dialogic reading. Early Child Res. Q. 15, 75–90. doi: 10.1016/S0885-2006(99)00038-1

[ref35] Harris P. L. de Rosnay M. Pons F. (2016). “Understanding emotions” in Handbook of emotions. eds. Feldman Barrett L. Lewis M. Haviland-Jones J. M. (New York, NY: Guilford Press), 293–306.

[ref36] Hindman A. H. Skibbe L. E. Foster T. D. (2014). Exploring the variety of parental talk during shared book reading and its contributions to preschool language and literacy: evidence from the early childhood longitudinal study-birth cohort. Read. Writ. 27, 287–313. doi: 10.1007/s11145-013-9445-4

[ref37] Hoffman J. Teale W. H. Yokota J. (2015). The book matters! Choosing narrative children’s literature to support read aloud discussion of complex texts in the early grades. Young Child. 70, 8–15. https://www.jstor.org/stable/ycyoungchildren.70.4.8

[ref38] Kılıç Ş. Hernandez Acton E. Zhu D. Dunsmore J. C. (2025). Parental emotion socialization and children’s emotional skills and socio-emotional functioning in early childhood in Türkiye and the United States. J. Genet. Psychol. 1–20, 1–20. doi: 10.1080/00221325.2025.2454314, PMID: 39921521

[ref39] Larson J. (2015). Children's services today: a practical guide for librarians. Lanham, MD: Rowman and Littlefield.

[ref40] Mar R. A. Oatley K. Djikic M. Mullin J. (2011). Emotion and narrative fiction: interactive influences before, during, and after reading. Cognit. Emot. 25, 818–833. doi: 10.1080/02699931.2010.515151, PMID: 21824023

[ref41] Mar R. A. Oatley K. Peterson J. B. (2009). Exploring the link between reading fiction and empathy: ruling out individual differences and examining outcomes. Communications 34, 407–428. doi: 10.1515/COMM.2009.025

[ref42] Misailidi P. Papoudi D. Brouzos A. (2013). Mind what teachers say: kindergarten teachers’ use of mental state language during picture story narration. Early Educ. Dev. 24, 1161–1174. doi: 10.1080/10409289.2013.765787

[ref43] Mol S. E. Bus A. G. De Jong M. T. Smeets D. J. (2008). Added value of dialogic parent–child book readings: a meta-analysis. Early Educ. Dev. 19, 7–26. doi: 10.1080/10409280701838603

[ref44] Moschovaki E. Meadows S. (2005). Young children's spontaneous participation during classroom book reading: differences according to various types of books. Early Child. Res. Pract. 7.

[ref9002] Murray L. Rayson H. Ferrari P. F. Wass S. V. Cooper P. J. (2022). Dialogic book-sharing as a privileged intersubjective space. Frontiers in psychology, 13:786991 35310233 10.3389/fpsyg.2022.786991 PMC8927819

[ref45] Oatley K. (2016). Fiction: simulation of social worlds. Trends Cogn. Sci. 20, 618–628. doi: 10.1016/j.tics.2016.06.002 27449184

[ref46] Ornaghi V. Agliati A. De Salvia G. Spampinato A. (2024). La lettura dialogica favorisce lo sviluppo psicologico dei bambini: presentazione di uno strumento. Bambini 1, 14–18.

[ref47] Ornaghi V. Brazzelli E. Grazzani I. Agliati A. Lucarelli M. (2017). Does training toddlers in emotion knowledge lead to changes in their prosocial and aggressive behavior toward peers at nursery? Early Educ. Dev. 28, 396–414. doi: 10.1080/10409289.2016.1238674

[ref48] Ornaghi V. Brockmeier J. Grazzani I. (2011). The role of language games in children’s understanding of mental states: a training study. J. Cogn. Dev. 12, 239–259. doi: 10.1080/15248372.2011.563487

[ref49] Ornaghi V. Grazzani I. Cherubin E. Conte E. Piralli F. (2015). ‘Let’s talk about emotions!’. The effect of conversational training on preschoolers’ emotion comprehension and prosocial orientation. Soc. Dev. 24, 166–183. doi: 10.1111/sode.12091

[ref50] Price L. H. Van Kleeck A. Huberty C. J. (2009). Talk during book sharing between parents and preschool children: a comparison between storybook and expository book conditions. Read. Res. Q. 44, 171–194. doi: 10.1598/RRQ.44.2.4

[ref51] Reddy V. (2008). How infants know minds. Cambridge, MA: Harvard University Press.

[ref52] Rezzonico S. Hipfner-Boucher K. Milburn T. Weitzman E. Greenberg J. Pelletier J.. (2015). Improving preschool educators' interactive shared book reading: effects of coaching in professional development. Am. J. Speech Lang. Pathol. 24, 717–732. doi: 10.1044/2015_AJSLP-14-0188 26363186

[ref53] Saarni C. (2008). “The interface of emotional development with social context” in Handbook of emotions. eds. Lewis M. Haviland-Jones J. M. Barrett L. F.. 3rd ed (New York, NY: The Guilford Press), 332–347.

[ref54] Schapira R. (2024) A shared book-reading intervention program to promote preschoolers’ empathy—program. Presented at the conference EARLI SIG 5 and SIG 28 Conference, University of Warsaw, Faculty of Education, Warsaw, Poland.

[ref55] Schapira R. Aram D. (2020). Shared book reading at home and preschoolers’ socio-emotional competence. Early Educ. Dev. 31, 819–837. doi: 10.1080/10409289.2019.1692624

[ref56] Schapira R. Bergman Deitcher D. Aram D. (2021). Variability and stability in parent–child discourse during and following repeated shared book reading. Read. Writ. 34, 273–300. doi: 10.1007/s11145-020-10072-y

[ref57] Schapira R. Bergman Deitcher D. Knafo-Noam A. (2024). Promoting empathy in preschools through shared book reading—an intervention program. Presentation at the conference of 27th biennial meeting of ISSBD, Lisbon, Portugal. Available online at: https://2024biennial.issbd.org/full-program-guide/, p. 312.

[ref58] Schmidt A. C. Pierce-Rivera M. van Huisstede L. Marley S. C. Bernstein K. A. Millinger J.. (2024). What’s the story with Storytime? An examination of preschool teachers’ drama-based and shared reading practices during picture book read-aloud. Early Child. Educ. J. 52, 1525–1543. doi: 10.1007/s10643-023-01554-z

[ref59] Sukhera J. (2022). Narrative reviews: flexible, rigorous and practical. J. Grad. Med. Educ. 14, 414–417. doi: 10.4300/JGME-D-22-00480.1, PMID: 35991099 PMC9380636

[ref60] Trevarthen C. (1993). “The self born in intersubjectivity: the psychology of an infant communicating” in The perceived self: ecological and interpersonal sources of self-knowledge. ed. Neisser U. (Cambridge: Cambridge University Press), 121–173.

[ref61] Voltmer K. von Salisch M. (2022). The feeling thinking talking intervention with teachers advances young children's emotion knowledge. Soc. Dev. 31, 846–861. doi: 10.1111/sode.12586

[ref62] Vygotsky L. S. (1978). Mind in society. Cambridge, MA: Harvard University Press.

[ref64] Wasik B. A. Bond M. A. (2001). Beyond the pages of a book: interactive book reading and language development in preschool classrooms. J. Educ. Psychol. 93, 243–250. doi: 10.1037/0022-0663.93.2.243

[ref65] Wasik B. A. Hindman A. H. (2020). Increasing preschoolers’ vocabulary development through a streamlined teacher professional development intervention. Early Child Res. Q. 50, 101–113. doi: 10.1016/j.ecresq.2018.11.001

[ref66] Whitehurst G. J. Arnold D. S. Epstein J. N. Angell A. L. Smith M. Fischel J. E. (1994). A picture book reading intervention in day care and home for children from low-income families. Dev. Psychol. 30, 679–689. doi: 10.1037/0012-1649.30.5.679

[ref67] Whitehurst G. J. Falco F. L. Lonigan C. J. Fischel J. E. DeBaryshe B. D. Valdez-Menchaca M. C.. (1988). Accelerating language development through picture book reading. Dev. Psychol. 24, 552–559. doi: 10.1037/0012-1649.24.4.552

[ref68] Zucker T. A. Cabell S. Q. Oh Y. Wang X. (2020). Asking questions is just the first step: using upward and downward scaffolds. Read. Teach. 74, 275–283. doi: 10.1002/trtr.1943

